# Soliton solutions for the Zoomeron model applying three analytical techniques

**DOI:** 10.1371/journal.pone.0283594

**Published:** 2023-07-27

**Authors:** Mohammad Safi Ullah, Md. Mostafa, M. Zulfikar Ali, Harun-Or Roshid, Mahinur Akter

**Affiliations:** 1 Department of Mathematics, Comilla University, Cumilla, Bangladesh; 2 Department of Mathematics, Pabna University of Science and Technology, Pabna, Bangladesh; 3 Department of Mathematics, University of Rajshahi, Rajshahi, Bangladesh; China University of Mining and Technology, CHINA

## Abstract

The Zoomeron equation is used in various categories of soliton with unique characteristics that arise in different physical phenomena, such as fluid dynamics, laser physics, and nonlinear optics. To achieve soliton solutions for the Zoomeron nonlinear structure, we apply the unified, the Kudryashov, and the improved Kudryashov techniques. We find periodic, breather, kink, anti-kink, and dark-bell soliton solutions from the derived optical soliton solutions. Bright, dark, and bright-dark breather waves are also established. Finally, some dynamic properties of the acquired findings are displayed in 3D, density, and 2D views.

## 1 Introduction

Nowadays, nonlinear models are the most established in the modern world for developing many complicated sciences such as mathematical modeling, fluid dynamics, electromagnetism, the atmosphere, weather forecasting, optics, and mathematical modeling. In the telecommunications industry, optical solitons are commonly used. The modern world directly depends on the concept of a solitary wave [1–8]. These types of problems can be solved using a variety of techniques. The ansatz and sub-equation theories [9], the ð-dressing method [10], the *tan*(*Θ*/2) expansion scheme [11], the Hirota scheme [12–15], the MSE method [16], the unified method [17], Riemann-Hilbert problem [18], ð-steepest descent method [19, 20] and other techniques [21–24] are commonly used to solve these problems. As a result of the development of the concept of soliton, many kinds of nonlinear models have been developed, such as the KP hierarchy model [25], the geophysical KdV structure [26], the KDV-Burger’s system [27], the Jimbo-Miwa nonlinear system [28], the Zoomeron model [29], and others. Among these models, the Zoomeron nonlinear structure is one of the incognito evolution models and was first introduced in 1976 by Calogero and Degasperis [30]. This equation supports various categories of solitons with unique characteristics that arise in different physical phenomena, such as fluid dynamics, laser physics, and nonlinear optics [31].

Our main objective in this investigation is to obtain some soliton solutions that show how the Zoomeron equation is physically constructed by the unified method [32, 33], the Kudryashov scheme [34], and the improved Kudryashov technique [35]. All hyperbolic tangent function approaches are combined into one technique known as the unified method [32]. This method also derives from two renowned methods, developed by Akkagil and Aydemir under the names *tanh* function and (*G*^′^/*G*) expansion [33]. In 2022, Ullah and his research team discovered the singular solution to the linear partial differential structure [36]. Additionally, in 2022, this technique was employed to resolve the Biswas-Arshed PDE model [37]. There has been no analysis of the soliton solution for the Zoomeron model using these methods. Moreover, the suggested combination can handle nonlinear problems of a versatile physical nature. The efficacy of the investigated techniques is demonstrated by the numerical results, which are highly positive.

## 2 ODE form of the Zoomeron model

The nonlinear Zoomeron equation’s precise solitary wave solution is expressed by the following formula:

$$\begin{array}{ccc} & & {\left(\frac{P_{xy}}{P}\right)}_{tt}-{\left(\frac{P_{xy}}{P}\right)}_{xx}+2{\left(P^{2}\right)}_{xt}=0, \end{array}$$ (1)

Here, *P*(*x*, *y*, *t*) is the amplitude portion of soliton, with position variables *x*, *y*, and time variable *t*. Eq (1) is reduced via the relation *P*(*x*, *y*, *t*) = *P*(*ζ*), *ζ* = *x* + *ry* − *mt* to the following ODE:

$$\begin{array}{ccc} & & r\left(m^{2}-1\right)P^{\prime \prime }-2mP^{3}-qP=0 \end{array}$$ (2)

## 3 Solution procedure for the unified scheme with its application

Now, taking the subsequent formal solution of Eq (1) with an auxiliary equation

$$\begin{array}{ccc} & & P\left(\zeta \right)=\sum_{j=0}^{K}A_{j}\lambda {\left(\zeta \right)}^{j}+\sum_{j=1}^{K}A_{-j}\lambda {\left(\zeta \right)}^{-j}, \end{array}$$ (3)

$$\begin{array}{ccc} & & \lambda^{\prime }\left(\zeta \right)=\lambda^{2}\left(\zeta \right)+\alpha . \end{array}$$ (4)

Eq (3) gives us 9 types of solutions in 3 families:

**Family-01:** Hyperbolic function (when *α* is less than zero):

$$\begin{array}{c} \lambda \left(\zeta \right)=\{\begin{array}{c} \frac{\sqrt{-(L^{2}+M^{2})\alpha }-L\sqrt{-\alpha }cosh\left(2\sqrt{-\alpha }\left(\zeta +\delta \right)\right)}{Lsinh(2\sqrt{-\alpha }\left(\zeta +\delta \right))+M},\\ \frac{-\sqrt{-(L^{2}+M^{2})\alpha }-L\sqrt{-\alpha }cosh\left(2\sqrt{-\alpha }\left(\zeta +\delta \right)\right)}{Lsinh(2\sqrt{-\alpha }\left(\zeta +\delta \right))+M},\\ \sqrt{-\alpha }+\frac{2L\sqrt{-\alpha }}{L+cosh\left(2\sqrt{-\alpha }\left(\zeta +\delta \right)\right)-sinh\left(2\sqrt{-\alpha }\left(\zeta +\delta \right)\right)},\\ -\sqrt{-\alpha }+\frac{2L\sqrt{-\alpha }}{L+cosh\left(2\sqrt{-\alpha }\left(\zeta +\delta \right)\right)-sinh\left(2\sqrt{-\alpha }\left(\zeta +\delta \right)\right)}, \end{array} \end{array}$$ (5)

**Family-02:** Trigonometric function (when *α* is greater than zero)

$$\begin{array}{c} \lambda \zeta )=\{\begin{array}{c} \frac{\sqrt{(L^{2}-M^{2})\alpha }-L\sqrt{\alpha }cos\left(2\sqrt{\alpha }\left(\zeta +\delta \right)\right)}{Lsin(2\sqrt{\alpha }\left(\zeta +\delta \right))+M},\\ \frac{-\sqrt{(L^{2}-M^{2})\alpha }-L\sqrt{\alpha }cos\left(2\sqrt{\alpha }\left(\zeta +\delta \right)\right)}{Lsin(2\sqrt{\alpha }\left(\zeta +\delta \right))+M},\\ i\sqrt{\alpha }+\frac{-2iL\sqrt{\alpha }}{L+cos\left(2\sqrt{\alpha }\left(\zeta +\delta \right)\right)-isin\left(2\sqrt{\alpha }\left(\zeta +\delta \right)\right)},\\ -i\sqrt{\alpha }+\frac{2iL\sqrt{\alpha }}{L+cos\left(2\sqrt{\alpha }\left(\zeta +\delta \right)\right)-isin\left(2\sqrt{\alpha }\left(\zeta +\delta \right)\right)}, \end{array} \end{array}$$ (6)

**Family-03:** Rational function (when *α* is equal to zero)

$$\begin{array}{c} \lambda \left(\zeta \right)=-\frac{1}{\zeta +\delta }, \end{array}$$ (7)

when *L* ≠ 0, *α*, and *M* are random parameters.

Using the balancing principle, *P*^3^ and *P*′′ result in *K* = 1. By changing the value of *K* in equation Eq (3), we have

$$\begin{array}{ccc} & & P\left(\zeta \right)=A_{0}+A_{1}\lambda \left(\zeta \right)+A_{-1}\lambda {\left(\zeta \right)}^{-1}. \end{array}$$ (8)

Putting Eqs (8) and (4) in Eq (2) and some simple calculation, we obtain the following three solutions set as

$$\begin{array}{c} \alpha =\frac{1}{2}\frac{q}{mA{{}_{1}}^{2}},m=m,r=\frac{mA_{1}^{2}}{m^{2}-1},A_{-1}=0,A_{0}=0,A_{1}=A_{1}, \end{array}$$ (9)

$$\begin{array}{c} \alpha =\frac{2mA{{}_{1}}^{2}}{q},m=m,r=\frac{1}{4}\frac{q^{2}}{mA_{-1}^{2}\left(m^{2}-1\right)},A_{-1}=A_{-1},A_{0}=0,A_{1}=0, \end{array}$$ (10)

$$\begin{array}{c} \alpha =\frac{1}{8}\frac{q}{mA{{}_{1}}^{2}},m=m,r=\frac{mA{{}_{1}}^{2}}{m^{2}-1},A_{-1}=-\frac{1}{8}\frac{q}{mA{}_{1}},A{}_{0}=0,A_{1}=A_{1}. \end{array}$$ (11)

Now, applying Eqs (5)–(8) and (9), now we get 9 types of exact solutions Eq (1) as below

$$\begin{array}{ccc} & & \;P_{11}\left(x,y,t\right)=A_{1}\left(\frac{\sqrt{-(L^{2}+M^{2})\alpha }-L\sqrt{-\alpha }cosh(2\sqrt{-\alpha }\left(\zeta +\delta \right)}{Lsinh(2\sqrt{-\alpha }\left(\zeta +\delta \right))+M}\right),\\ & & \;P_{12}\left(x,y,t\right)=A_{1}\left(\frac{-\sqrt{-(L^{2}+M^{2})\alpha }-L\sqrt{-\alpha }cosh\left(2\sqrt{-\alpha }\left(\zeta +\delta \right)\right)}{Lsinh(2\sqrt{-\alpha }\left(\zeta +\delta \right))+M}\right),\\ & & \;P_{13}\left(x,y,t\right)=A_{1}\left(\sqrt{-\alpha }+\frac{2L\sqrt{-\alpha }}{L+cosh\left(2\sqrt{-\alpha }\left(\zeta +\delta \right)\right)-sinh\left(2\sqrt{-\alpha }\left(\zeta +\delta \right)\right)}\right),\\ & & \;P_{14}\left(x,y,t\right)=A_{1}\left(-\sqrt{-\alpha }+\frac{2L\sqrt{-\alpha }}{L+cosh\left(2\sqrt{-\alpha }\left(\zeta +\delta \right)\right)-sinh\left(2\sqrt{-\alpha }\left(\zeta +\delta \right)\right)}\right),\\ & & \;P_{15}\left(x,y,t\right)=A_{1}\left(\frac{\sqrt{(L^{2}-M^{2})\alpha }-L\sqrt{\alpha }cos\left(2\sqrt{\alpha }\left(\zeta +\delta \right)\right)}{Lsin(2\sqrt{\alpha }\left(\zeta +\delta \right))+M}\right),\\ & & \;P_{16}\left(x,y,t\right)=A_{1}\left(\frac{-\sqrt{(L^{2}-M^{2})\alpha }-L\sqrt{\alpha }cos\left(2\sqrt{\alpha }\left(\zeta +\delta \right)\right)}{Lsin(2\sqrt{\alpha }\left(\zeta +\delta \right))+M}\right),\\ & & \;P_{17}\left(x,y,t\right)=A_{1}\left(i\sqrt{\alpha }+\frac{-2iL\sqrt{\alpha }}{L+cos\left(2\sqrt{\alpha }\left(\zeta +\delta \right)\right)-isin\left(2\sqrt{\alpha }\left(\zeta +\delta \right)\right)}\right),\\ & & \;P_{18}\left(x,y,t\right)=A_{1}\left(-i\sqrt{\alpha }+\frac{2iL\sqrt{\alpha }}{L+cos\left(2\sqrt{\alpha }\left(\zeta +\delta \right)\right)-isin\left(2\sqrt{\alpha }\left(\zeta +\delta \right)\right)}\right),\\ & & \;P_{19}\left(x,y,t\right)=A_{1}\left(\frac{-1}{\zeta +\delta }\right), \end{array}$$

where $\alpha =\frac{1}{2}\frac{q}{mA{{}_{1}}^{2}},$ and $r=\frac{mA_{1}^{2}}{\left(m-1)(m+1\right)}.$

Again, applying Eqs (5)–(8) and (10), we get 9 types of exact solutions Eq (1) as below.

$$\begin{array}{ccc} & & \;P_{21}\left(x,y,t\right)={\left(\frac{\sqrt{-(L^{2}+M^{2})\alpha }-L\sqrt{-\alpha }cosh\left(2\sqrt{-\alpha }\left(\zeta +\delta \right)\right)}{Lsinh(2\sqrt{-\alpha }\left(\zeta +\delta \right))+M}\right)}^{-1}A_{-1},\\ & & \;P_{22}\left(x,y,t\right)={\left(\frac{-\sqrt{-(L^{2}+M^{2}))\alpha }-L\sqrt{-\alpha }cosh\left(2\sqrt{-\alpha }\left(\zeta +\delta \right)\right)}{Lsinh(2\sqrt{-\alpha }\left(\zeta +\delta \right))+M}\right)}^{-1}A_{-1},\\ & & \;P_{23}\left(x,y,t\right)={\left(\sqrt{-\alpha }+\frac{2L\sqrt{-\alpha }}{L+cosh\left(2\sqrt{-\alpha }\left(\zeta +\delta \right)\right)-sinh\left(2\sqrt{-\alpha }\left(\zeta +\delta \right)\right)}\right)}^{-1}A_{-1},\\ & & \;P_{24}\left(x,y,t\right)={\left(-\sqrt{-\alpha }+\frac{2L\sqrt{-\alpha }}{L+cosh\left(2\sqrt{-\alpha }\left(\zeta +\delta \right)\right)-sinh\left(2\sqrt{-\alpha }\left(\zeta +\delta \right)\right)}\right)}^{-1}A_{-1},\\ & & \;P_{25}\left(x,y,t\right)={\left(\frac{\sqrt{(L^{2}-M^{2})\alpha }-L\sqrt{\alpha }cos\left(2\sqrt{\alpha }\left(\zeta +\delta \right)\right)}{Lsin(2\sqrt{\alpha }\left(\zeta +\delta \right))+M}\right)}^{-1}A_{-1},\\ & & \;P_{26}\left(x,y,t\right)={\left(\frac{-\sqrt{(L^{2}-M^{2})\alpha }-L\sqrt{\alpha }cos\left(2\sqrt{\alpha }\left(\zeta +\delta \right)\right)}{Lsin(2\sqrt{\alpha }\left(\zeta +\delta \right))+M}\right)}^{-1}A_{-1},\\ & & \;P_{27}\left(x,y,t\right)={\left(i\sqrt{\alpha }+\frac{-2iL\sqrt{\alpha }}{L+cos\left(2\sqrt{\alpha }\left(\zeta +\delta \right)\right)-isin\left(2\sqrt{\alpha }\left(\zeta +\delta \right)\right)}\right)}^{-1}A_{-1},\\ & & \;P_{28}\left(x,y,t\right)={\left(-i\sqrt{\alpha }-\frac{2iL\sqrt{\alpha }}{L+cos\left(2\sqrt{\alpha }\left(\zeta +\delta \right)\right)-isin\left(2\sqrt{\alpha }\left(\zeta +\delta \right)\right)}\right)}^{-1}A_{-1},\\ & & \;P_{29}\left(x,y,t\right)={\left(-\frac{1}{\zeta +\delta }\right)}^{-1}A_{-1}, \end{array}$$

where $\alpha =\frac{2mA{{}_{1}}^{2}}{q},$ and $r=\frac{1}{4}\frac{q^{2}}{mA_{-1}^{2}\left(m-1\right)\left(m+1\right)}.$

Again, applying Eqs (11) and (6)–(8), we get 9 types of exact solutions Eq (1) as below.

$$\begin{array}{ccc} & & \;P_{31}\left(x,y,t\right)=A_{1}\left(\frac{\sqrt{-(L^{2}+M^{2})\alpha }-L\sqrt{-\alpha }cosh\left(2\sqrt{-\alpha }\left(\zeta +\delta \right)\right)}{Lsinh(2\sqrt{-\alpha }\left(\zeta +\delta \right))+M}\right)\\ & & \;\;\;\;\;\;+A_{-1}{\left(\frac{\sqrt{-(L^{2}+M^{2})\alpha }-L\sqrt{-\alpha }cosh\left(2\sqrt{-\alpha }\left(\zeta +\delta \right)\right)}{Lsinh(2\sqrt{-\alpha }\left(\zeta +\delta \right))+M}\right)}^{-1},\\ & & \;P_{32}\left(x,y,t\right)=A_{1}\left(\frac{-\sqrt{-(L^{2}+M^{2})\alpha }-L\sqrt{-\alpha }cosh\left(2\sqrt{-\alpha }\left(\zeta +\delta \right)\right)}{Lsinh(2\sqrt{-\alpha }\left(\zeta +\delta \right))+M}\right)\\ & & \;\;\;\;\;\;+A_{-1}{\left(\frac{-\sqrt{-(L^{2}+M^{2})\alpha }-L\sqrt{-\alpha }cosh\left(2\sqrt{-\alpha }\left(\zeta +\delta \right)\right)}{Lsinh(2\sqrt{-\alpha }\left(\zeta +\delta \right))+M}\right)}^{-1},\\ & & \;P_{33}\left(x,y,t\right)=A_{1}\left(\sqrt{-\alpha }+\frac{2L\sqrt{-\alpha }}{L+cosh\left(2\sqrt{-\alpha }\left(\zeta +\delta \right)\right)-sinh\left(2\sqrt{-\alpha }\left(\zeta +\delta \right)\right)}\right)\\ & & \;\;\;\;\;\;+A_{-1}{\left(\sqrt{-\alpha }+\frac{2L\sqrt{-\alpha }}{L+cosh\left(2\sqrt{-\alpha }\left(\zeta +\delta \right)\right)-sinh\left(2\sqrt{-\alpha }\left(\zeta +\delta \right)\right)}\right)}^{-1},\\ & & \;P_{34}\left(x,y,t\right)=A_{1}\left(-\sqrt{-\alpha }+\frac{2L\sqrt{-\alpha }}{L+cosh\left(2\sqrt{-\alpha }\left(\zeta +\delta \right)\right)-sinh\left(2\sqrt{-\alpha }\left(\zeta +\delta \right)\right)}\right)\\ & & \;\;\;\;\;\;+A_{-1}{\left(-\sqrt{-\alpha }+\frac{2L\sqrt{-\alpha }}{L+cosh\left(2\sqrt{-\alpha }\left(\zeta +\delta \right)\right)-sinh\left(2\sqrt{-\alpha }\left(\zeta +\delta \right)\right)}\right)}^{-1},\\ & & \;P_{35}\left(x,y,t\right)=A_{1}\left(\frac{\sqrt{(L^{2}-M^{2})\alpha }-L\sqrt{\alpha }cos\left(2\sqrt{\alpha }\left(\zeta +\delta \right)\right)}{Lsin(2\sqrt{\alpha }\left(\zeta +\delta \right))+M}\right)\\ & & \;\;\;\;\;\;+A_{-1}{\left(\frac{\sqrt{(L^{2}-M^{2})\alpha }-L\sqrt{\alpha }cos\left(2\sqrt{\alpha }\left(\zeta +\delta \right)\right)}{Lsin(2\sqrt{\alpha }\left(\zeta +\delta \right))+M}\right)}^{-1},\\ & & \;P_{36}\left(x,y,t\right)=A_{1}\left(\frac{-\sqrt{(L^{2}-M^{2})\alpha }-L\sqrt{\alpha }cos\left(2\sqrt{\alpha }\left(\zeta +\delta \right)\right)}{Lsin(2\sqrt{\alpha }\left(\zeta +\delta \right))+M}\right)\\ & & \;\;\;\;\;\;+A_{-1}{\left(\frac{-\sqrt{(L^{2}-M^{2})\alpha }-L\sqrt{\alpha }cos\left(2\sqrt{\alpha }\left(\zeta +\delta \right)\right)}{Lsin(2\sqrt{\alpha }\left(\zeta +\delta \right))+M}\right)}^{-1},\\ & & \;P_{37}\left(x,y,t\right)=A_{1}\left(i\sqrt{\alpha }+\frac{-2iL\sqrt{\alpha }}{L+cos\left(2\sqrt{\alpha }\left(\zeta +\delta \right)\right)-isin\left(2\sqrt{\alpha }\left(\zeta +\delta \right)\right)}\right)\\ & & \;\;\;\;\;\;+A_{-1}{\left(i\sqrt{\alpha }+\frac{-2iL\sqrt{\alpha }}{L+cos\left(2\sqrt{\alpha }\left(\zeta +\delta \right)\right)-isin\left(2\sqrt{\alpha }\left(\zeta +\delta \right)\right)}\right)}^{-1},\\ & & \;P_{38}\left(x,y,t\right)=A_{1}\left(-i\sqrt{\alpha }-\frac{2iL\sqrt{\alpha }}{L+cos\left(2\sqrt{\alpha }\left(\zeta +\delta \right)\right)-isin\left(2\sqrt{\alpha }\left(\zeta +\delta \right)\right)}\right)\\ & & \;\;\;\;\;\;+A_{-1}{\left(-i\sqrt{\alpha }-\frac{2iL\sqrt{\alpha }}{L+cos\left(2\sqrt{\alpha }\left(\zeta +\delta \right)\right)-isin\left(2\sqrt{\alpha }\left(\zeta +\delta \right)\right)}\right)}^{-1},\\ & & \;P_{39}\left(x,y,t\right)=A_{1}\left(-\frac{1}{\zeta +\delta }\right)+A_{-1}{\left(-\frac{1}{\zeta +\delta }\right)}^{-1}, \end{array}$$

where $\zeta =x+ry-mt,\alpha =\frac{1}{8}\frac{q}{mA{{}_{1}}^{2}},$ and $r=\frac{mA{{}_{1}}^{2}}{\left(m-1)(m+1\right)}.$

## 4 Solution procedure for the Kudryashov scheme with its application

Here, we use the trial solution to Eq (1), which is expressed as a polynomial in λ(*ζ*)

$$\begin{array}{ccc} & & P\left(\zeta \right)=\sum_{i=0}^{K}A_{i}\lambda {\left(\zeta \right)}^{i}, \end{array}$$ (12)

$$\begin{array}{ccc} & & \lambda^{\prime }\left(\zeta \right)=\lambda^{2}\left(\zeta \right)-\lambda \left(\zeta \right), \end{array}$$ (13)

and λ is given by

$$\begin{array}{c} \lambda \left(\zeta \right)=\frac{1}{1+e^{\zeta }}. \end{array}$$ (14)

when *α* is an arbitrary parameter.

Using the balancing principle, *P*^3^ and *P*′′ result in *K* = 1. By changing the value of *K* in equation Eq (12), we have

$$\begin{array}{ccc} & & P\left(\zeta \right)=A_{0}+A_{1}\lambda \left(\zeta \right). \end{array}$$ (15)

Putting Eqs (15) and (13) in Eq (2) and some calculation, we obtain the following solution set as

$$\begin{array}{ccc} & & m=m,q=-2mA_{0}^{2},r=\frac{4A_{0}^{2}m}{\left(m^{2}-1\right)},A_{0}=A_{0},A_{1}=-2A_{0}. \end{array}$$ (16)

Now, applying Eqs (14), (15) and (16), occurs the subsequent analytic solution of Zoomeron equation:

$$\begin{array}{ccc} & & \;P_{40}\left(x,y,t\right)=A_{0}\left(1+\frac{2}{1+e^{\zeta }}\right), \end{array}$$

where *ζ* = *x* + *ry* − *mt*, and $r=\frac{4A_{0}^{2}m}{\left(m^{2}-1\right)}.$

## 5 Solution procedure for the improved Kudryashov scheme with its application

Now, the trial solution to Eq (1) is expressed by the next finite rational expansion:

$$\begin{array}{ccc} & & P\left(\zeta \right)=\frac{\sum_{i=0}^{K}A_{i}\lambda {\left(\zeta \right)}^{i}}{\sum_{j=1}^{L}B_{j}\lambda {\left(\zeta \right)}^{j}}, \end{array}$$ (17)

$$\begin{array}{ccc} & & \lambda^{\prime }\left(\zeta \right)=\alpha -\lambda^{2}\left(\zeta \right). \end{array}$$ (18)

Eq (18) gives five types of solutions as follows:

$$\begin{array}{c} \lambda \left(\zeta \right)=\{\begin{array}{c} \sqrt{\alpha }\text{tanh}\left(\sqrt{\alpha }\zeta \right),\;\;\;\;\alpha >0;\\ \sqrt{\alpha }\text{coth}\left(\sqrt{\alpha }\zeta \right),\;\;\;\;\alpha >0;\\ \;\;\;\frac{1}{\zeta },\;\;\;\;\;\;\;\alpha =0;\\ -\sqrt{-\alpha }\text{tan}\left(\sqrt{-\alpha }\zeta \right),\;\;\alpha <0;\\ \sqrt{-\alpha }\text{cot}\left(\sqrt{-\alpha }\zeta \right),\;\;\;\alpha <0; \end{array} \end{array}$$ (19)

when *α* is an arbitrary parameter.

Using the balancing principle, *P*^3^ and *P*′′ result in *K* = 1. By changing the value of *K* in equation Eq (17), we have

$$\begin{array}{ccc} & & P\left(\zeta \right)=\frac{A_{0}+A_{1}\lambda \left(\zeta \right)+A_{2}\lambda {\left(\zeta \right)}^{2}}{B_{0}+B_{1}\lambda \left(\zeta \right)}. \end{array}$$ (20)

Putting Eqs (20) and (18) in Eq (2) and some calculation, we obtain the following solution set as

$$\begin{array}{ccc} & & \alpha =\frac{\left(\frac{\left(-6m^{2}+6\right)B_{0}^{2}A_{2}^{2}m}{\left(m^{2}-1\right)B_{1}^{2}}+\frac{6mA_{2}^{2}B_{0}^{2}}{B_{1}^{2}}+B_{1}^{2}q\right)\left(m^{2}-1\right)}{\left(2m^{2}-2\right)A_{2}^{2}m},m=m,r=\frac{A_{2}^{2}m}{\left(m^{2}-1\right)B_{1}^{2}},\\ & & A_{0}=0,A_{1}=\frac{A_{2}B_{0}}{B_{1}},A_{2}=A_{2},B_{0}=B_{0},B_{1}=B_{1}. \end{array}$$ (21)

Now, applying Eqs (20) and (19) and (21), we get the 5 subsequent kinds of exact solutions to the Zoomeron model:

$$\begin{array}{ccc} & & \;P_{41}\left(x,y,t\right)=\frac{A_{1}\left(\sqrt{\alpha }\text{tanh}\left(\sqrt{\alpha }\zeta \right)\right)+A_{2}{\left(\sqrt{\alpha }\text{tanh}\left(\sqrt{\alpha }\zeta \right)\right)}^{2}}{B_{0}+B_{1}\left(\sqrt{\alpha }\text{tanh}\left(\sqrt{\alpha }\zeta \right)\right)},\\ & & \;P_{42}\left(x,y,t\right)=\frac{A_{1}\left(\sqrt{\alpha }\text{coth}\left(\sqrt{\alpha }\zeta \right)\right)+A_{2}{\left(\sqrt{\alpha }\text{coth}\left(\sqrt{\alpha }\zeta \right)\right)}^{2}}{B_{0}+B_{1}\left(\sqrt{\alpha }\text{coth}\left(\sqrt{\alpha }\zeta \right)\right)},\\ & & \;P_{43}\left(x,y,t\right)=\frac{A_{1}\left(\frac{1}{\zeta }\right)+A_{2}{\left(\frac{1}{\zeta }\right)}^{2}}{B_{0}+B_{1}\left(\frac{1}{\zeta }\right)},\\ & & \;P_{44}\left(x,y,t\right)=\frac{A_{1}\left(-\sqrt{-\alpha }\text{tan}\left(\sqrt{-\alpha }\zeta \right)\right)+A_{2}{\left(-\sqrt{-\alpha }\text{tan}\left(\sqrt{-\alpha }\zeta \right)\right)}^{2}}{B_{0}+B_{1}\left(-\sqrt{-\alpha }\text{tan}\left(\sqrt{-\alpha }\zeta \right)\right)},\\ & & \;P_{45}\left(x,y,t\right)=\frac{A_{1}\left(\sqrt{-\alpha }\text{cot}\left(\sqrt{-\alpha }\zeta \right)\right)+A_{2}{\left(\sqrt{-\alpha }\text{cot}\left(\sqrt{-\alpha }\zeta \right)\right)}^{2}}{B_{0}+B_{1}\left(\sqrt{-\alpha }\text{cot}\left(\sqrt{-\alpha }\zeta \right)\right)}, \end{array}$$

where $\alpha =\frac{\left(\frac{\left(-6m^{2}+6\right)B_{0}^{2}A_{2}^{2}m}{\left(m^{2}-1\right)B_{1}^{2}}+\frac{6mA_{2}^{2}B_{0}^{2}}{B_{1}^{2}}+B_{1}^{2}q\right)\left(m^{2}-1\right)}{\left(2m^{2}-2\right)A_{2}^{2}m},A_{1}=\frac{A_{2}B_{0}}{B_{1}},$ and $r=\frac{A_{2}^{2}m}{\left(m^{2}-1\right)B_{1}^{2}}.$

## 6 Figure analysis

**Fig 1:**
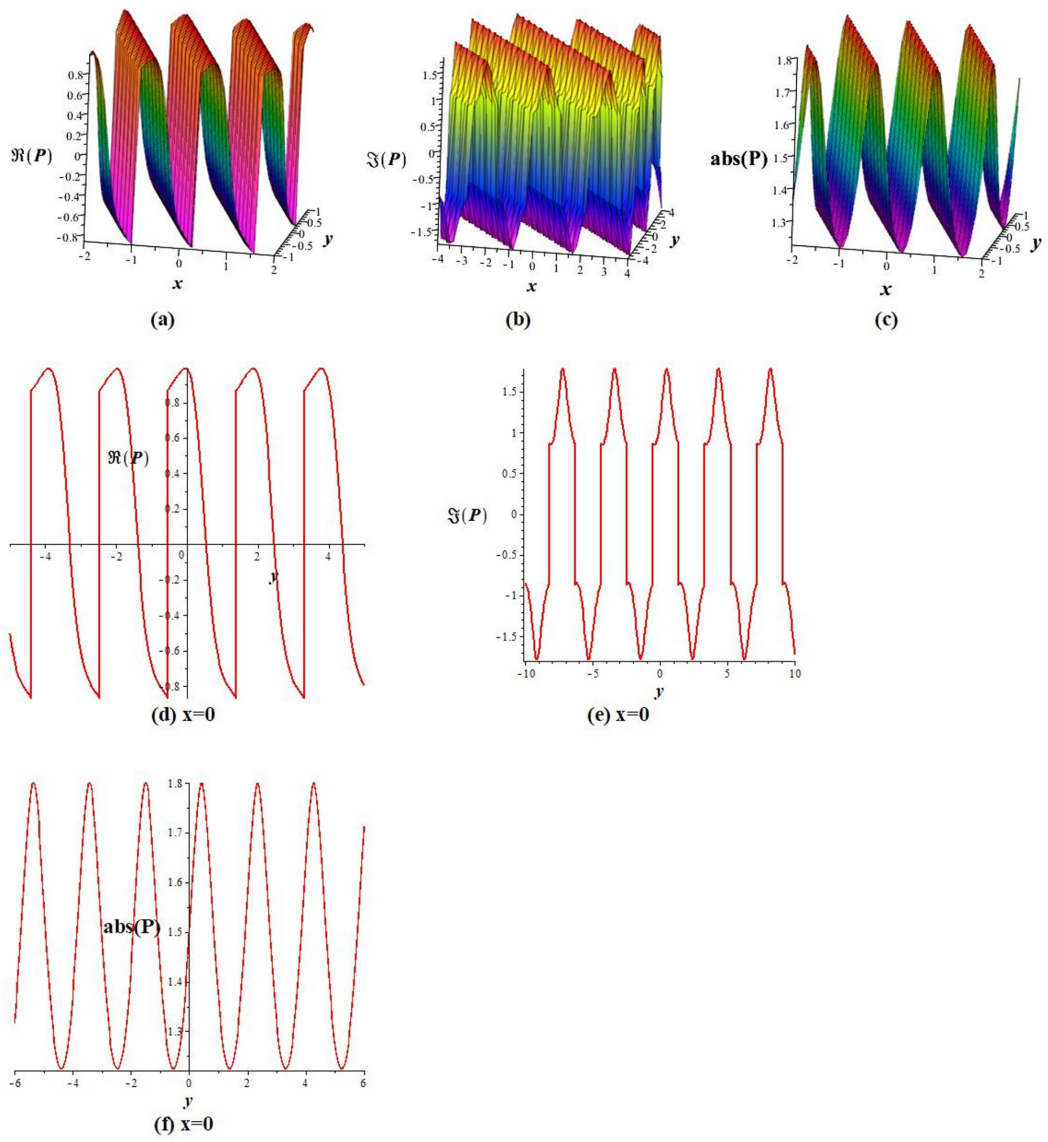
Graphical representation of *P*_11_ for *q* = 6, *m* = 2, *A*_0_ = 0, *A*_1_ = 1, *A*_−1_ = 0, *L* = *M* = *δ* = 1 at *t* = 0: (a-c) 3D shape, and (d-f) 2D shape.

**Fig 2:**
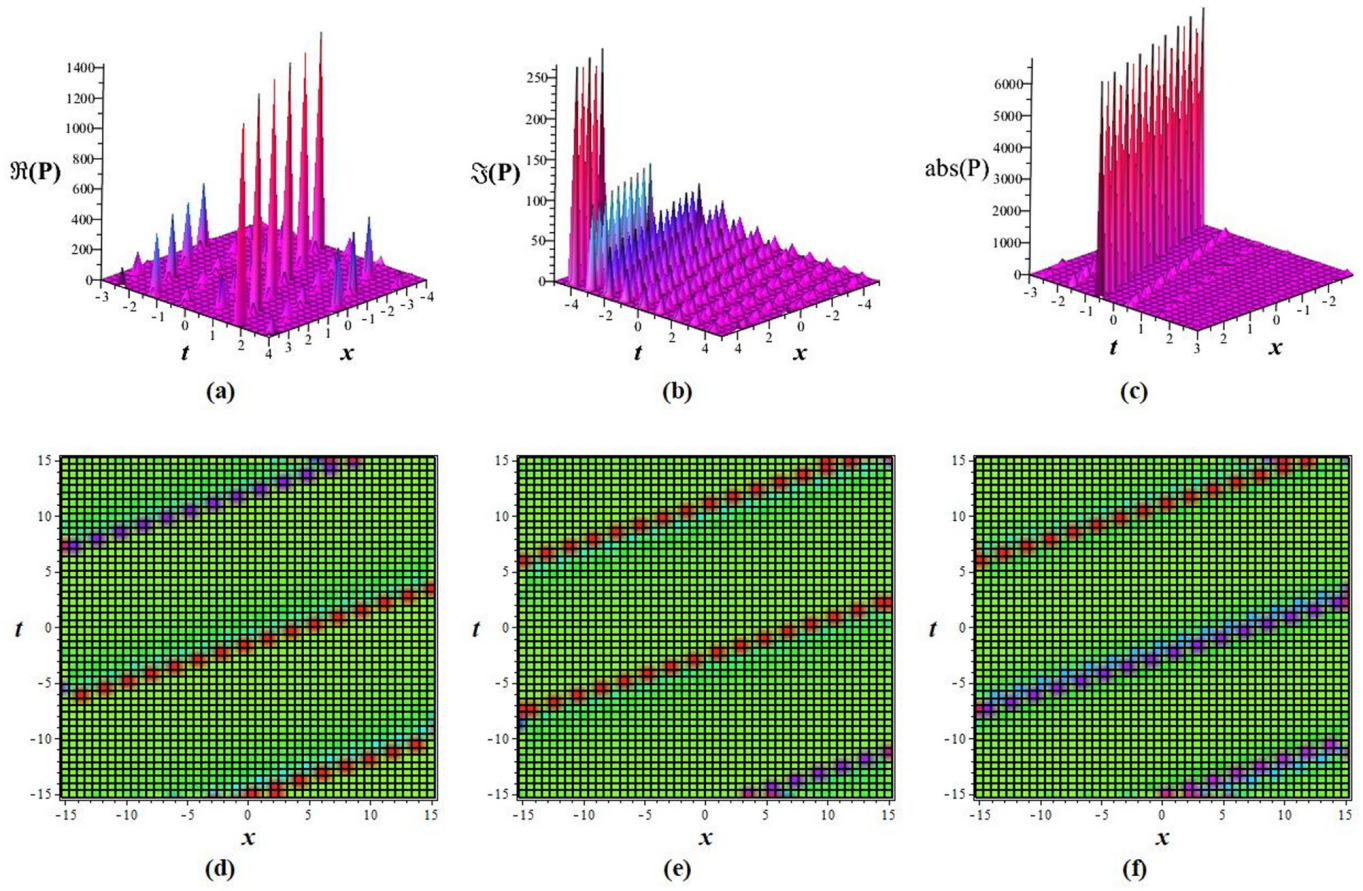
Wave pattern of *P*_15_ for *q* = 6, *m* = 3, *A*_0_ = 0, *A*_1_ = 1, *A*_−1_ = 0, *L* = *M* = *δ* = 1 at *y* = 0: (a-c) 3D view, and (d-f) density view.

**Fig 3:**
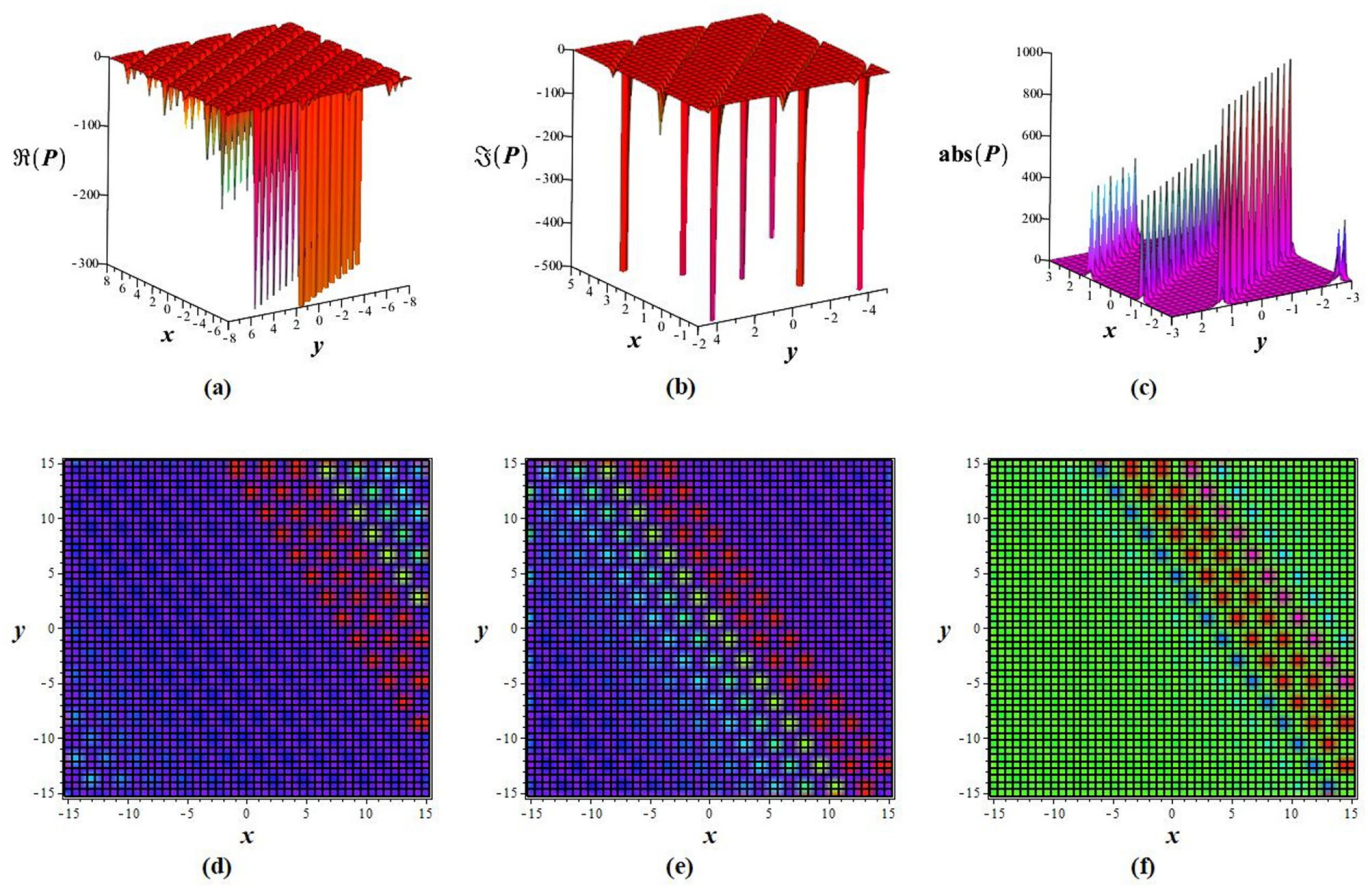
Wave pattern of *P*_16_ for *q* = 6, *m* = 2, *A*_0_ = 0, *A*_1_ = 1, *A*_−1_ = 0, *L* = *M* = *δ* = 1 at *t* = 0: (a-c) 3D view, and (d-f) density view.

**Fig 4:**
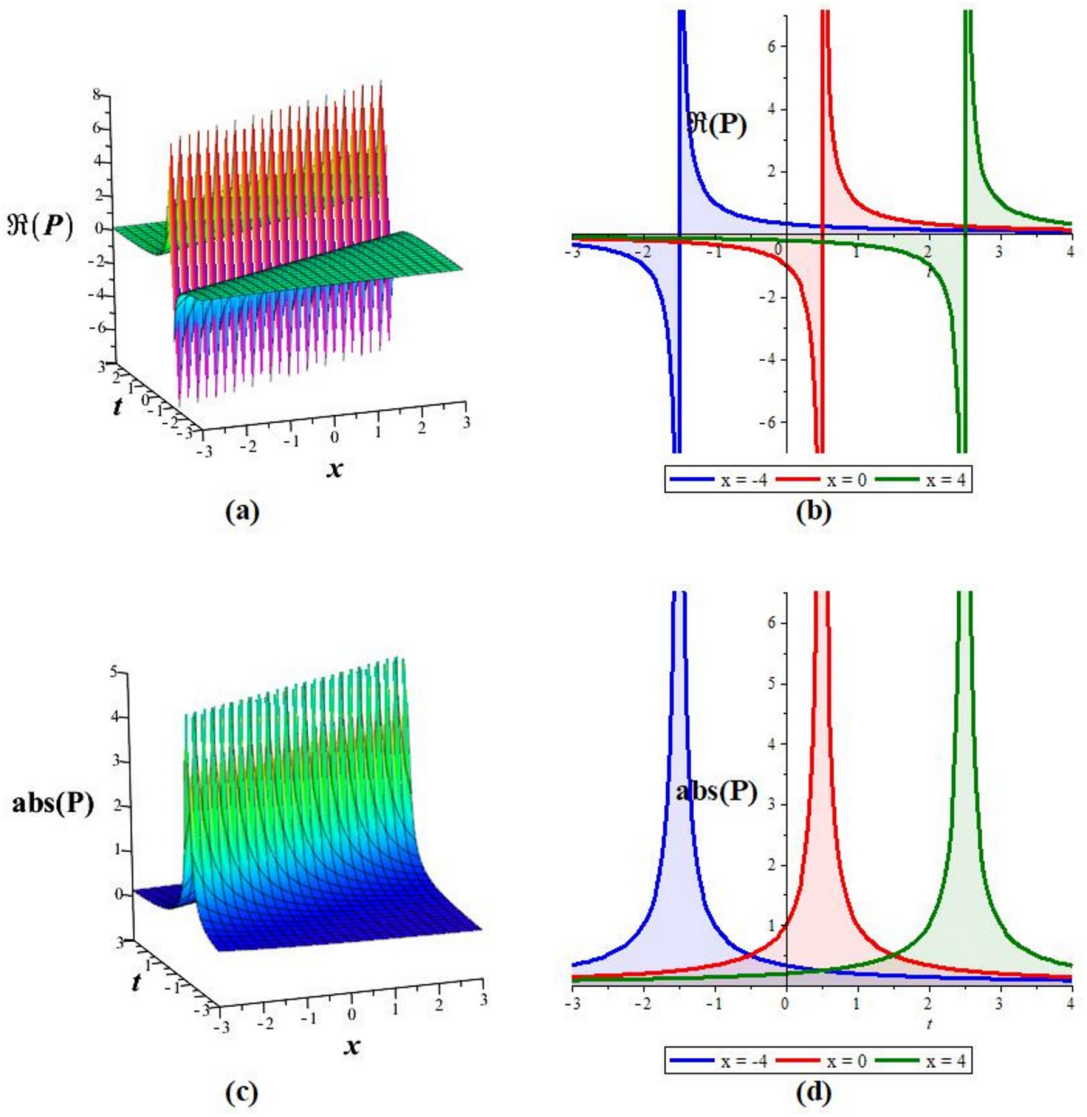
Graphical representation of *P*_19_ for *q* = 6, *m* = 2, *A*_0_ = 0, *A*_1_ = 1, *A*_−1_ = 0, *L* = *M* = *δ* = 1 at *y* = 0: (a, c) 3D wave pattern, and (b, d) 2D wave pattern.

**Fig 5:**
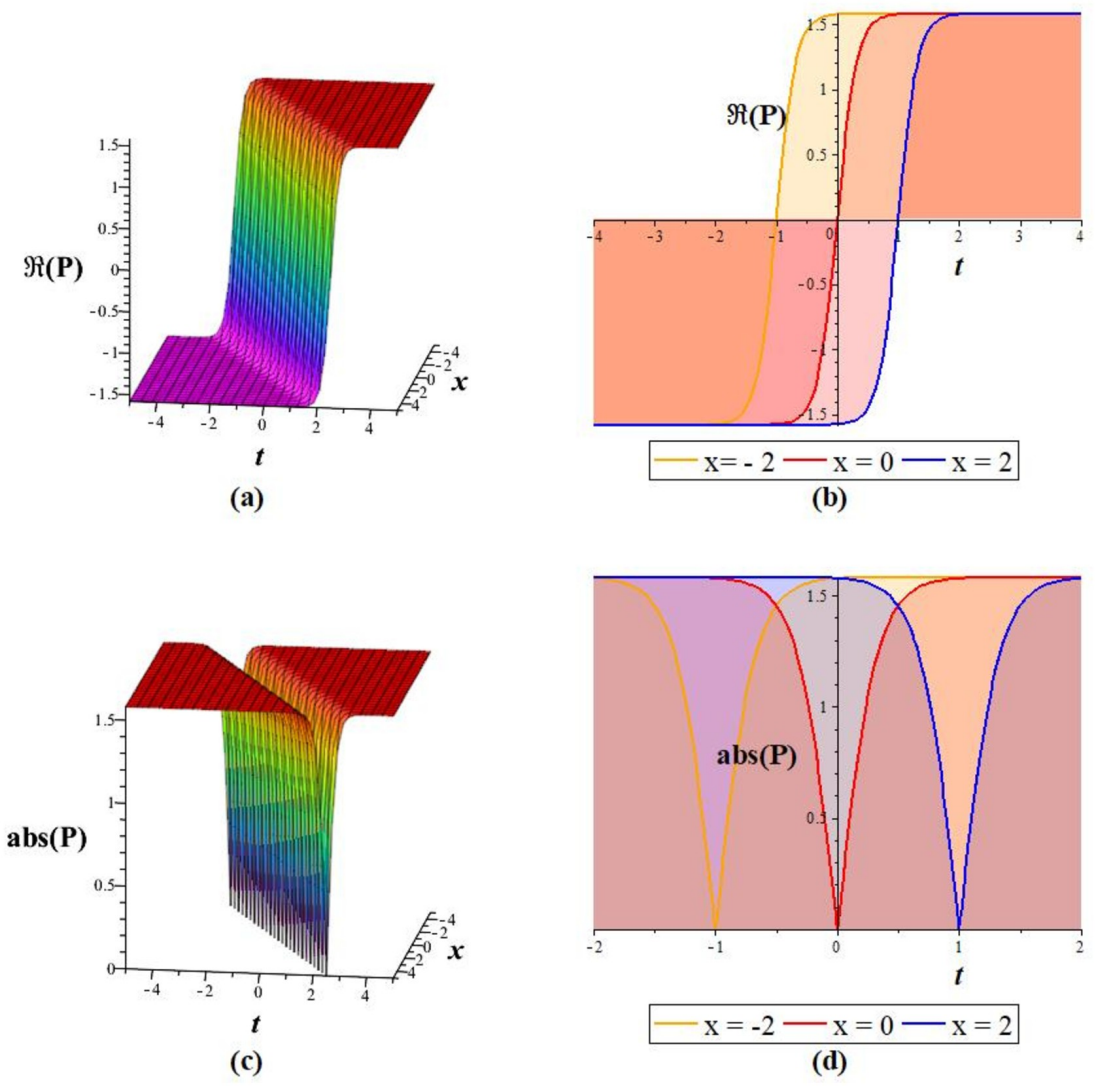
Graphical representation of *P*_44_ for *q* = 10, *m* = 2, *A*_0_ = *A*_−1_ = 0, *A*_1_ = *A*_2_ = *B*_0_ = *B*_1_ = 1 at *y* = 0: (a,c) 3D wave pattern, and (b,d) 2D wave pattern.

**Fig 6:**
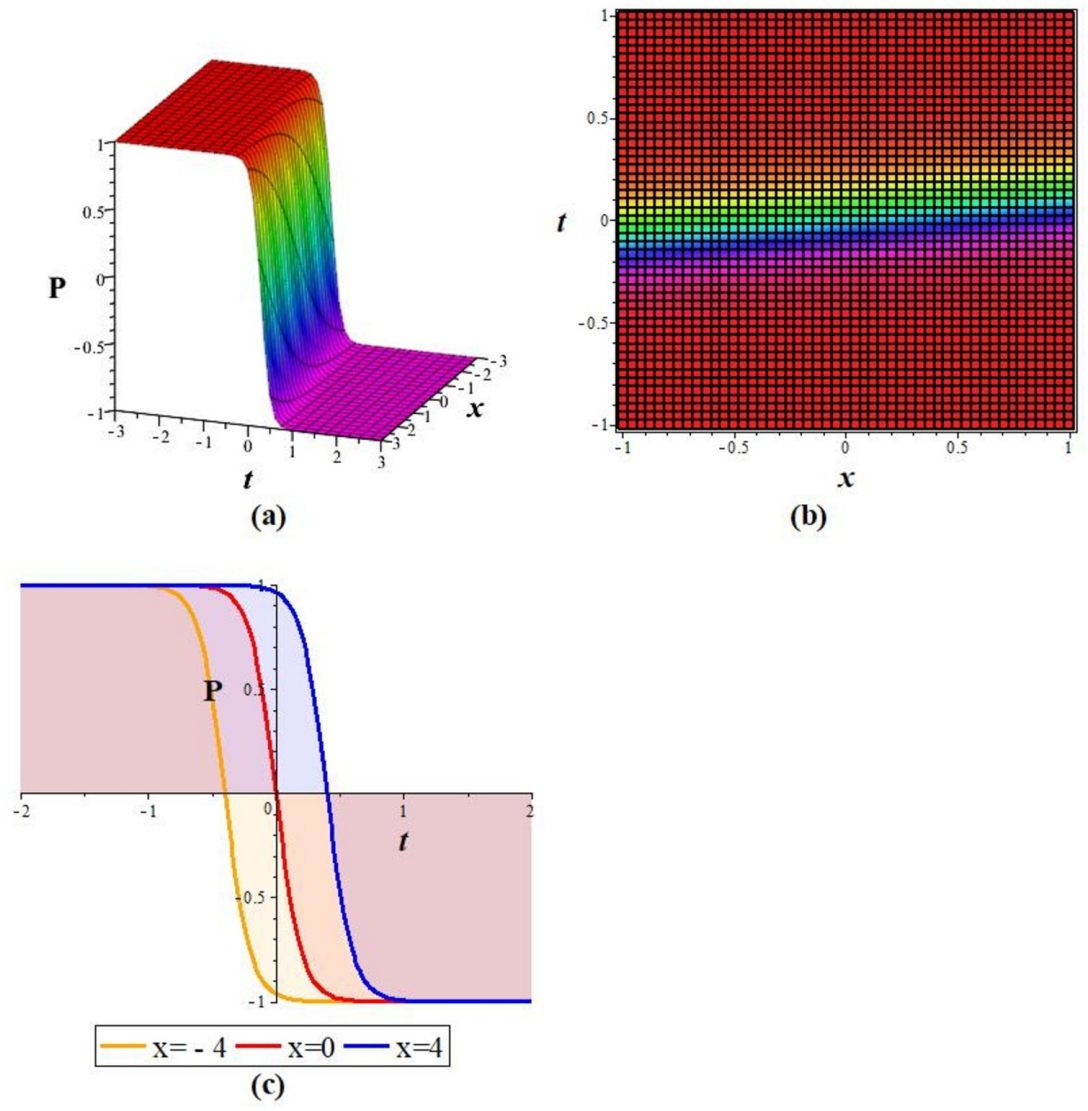
Graphical representation of *P*_40_ for *m* = 10, *A*_0_ = *L* = 1 at *y* = 0: (a) cubic pattern (b) density pattern, and (c) 2D plot.

Solutions *P*_11_, *P*_12_, *P*_21_, *P*_23_, *P*_22_, *P*_31_, and *P*_32_ exhibit a periodic wave, as drawn in Fig 1a–1f by *P*_11_. Solutions *P*_15_, *P*_25_, and *P*_35_ display a bright-breather wave, as plotted in Fig 2a–2f for *P*_15_. The same pattern is observed for solutions *P*_16_, *P*_26_, and *P*_36_, as shown in Fig 3a–3f for *P*_16_. The *Re*(*P*_16_) and *Im*(*P*_16_) represent the dark breather wave, and the absolute value plots of the solution *P*_16_ represent the bright breather wave. Breather wave property is also expressed by solutions *P*_19_, *P*_29_, *P*_39_, *P*_43_, and *P*_45_, as presented in Fig 4a–4d for *P*_19_. The *Re*(*P*_19_) gives a bright-dark breather wave, and the absolute value plot of the solution *P*_19_ expresses a bright breather wave solution. The *Re*(*P*_44_) displays an anti-kink wave, and the *abs*(*P*_44_) represents an anti-bell-shaped solitons solution, as depicted in Fig 5a–5d. Solution *P*_40_ gives a kink wave as illustrated in Fig 6a–6c.

## 7 Conclusion

We have successfully employed the unified, the Kudryashov, and the improved Kudryashov techniques to achieve soliton solutions for the Zoomeron model. We can observe periodic, breather, kink, anti-kink, and dark-bell soliton solutions in the derived optical soliton solutions. Bright, dark, and bright-dark breather waves are also established. Such solutions will play a vital role in further studies of the model.

## Supporting information

S1 File (PDF)

S2 File (PDF)
