# Supplementary material for: Soliton solutions for the Zoomeron model applying three analytical techniques
Source: PLoS One. 2023 Jul 27;18(7):e0283594. doi: 10.1371/journal.pone.0283594 (PMC10374063; doi:10.1371/journal.pone.0283594)
Supplement: S1 File — (PDF) [file pone.0283594.s001.pdf]

Funding

The authors received no specific funding for this work.

Corresponding Author: Mohammad Safi Ullah
Associate Professor
Dept. of Mathematics
Comilla University, Cumilla-3506
Email: safi.ru1985@gmail.com
